# Office-Visit Heart Rate and Long-Term Cardiovascular Events in Patients with Acute Myocardial Infarction

**DOI:** 10.3390/jcm12113734

**Published:** 2023-05-29

**Authors:** Jaeho Byeon, Eun Ho Choo, Ik Jun Choi, Kwan Yong Lee, Byung-Hee Hwang, Chan Joon Kim, Doo Soo Jeon, Youngkeun Ahn, Myung Ho Jeong, Kiyuk Chang

**Affiliations:** 1 Division of Cardiology, Department of Internal Medicine, Incheon St. Mary’s Hospital, College of Medicine, The Catholic University of Korea, Seoul 06591, Republic of Korea; teejh@naver.com (J.B.); mrfasthand@catholic.ac.kr (I.J.C.); jeondoosoo@hanmail.net (D.S.J.); 2 Division of Cardiology, Department of Internal Medicine, Seoul St. Mary’s Hospital, College of Medicine, The Catholic University of Korea, Seoul 06591, Republic of Korea; cmcchu@catholic.ac.kr (E.H.C.); cycle210@catholic.ac.kr (K.Y.L.); hbhmac@naver.com (B.-H.H.); 3 Division of Cardiology, Department of Internal Medicine, Uijeongbu St. Mary’s Hospital, College of Medicine, The Catholic University of Korea, Seoul 06591, Republic of Korea; godandsci@catholic.ac.kr; 4 Division of Cardiology, Department of Internal Medicine, Chonnam National University Hospital, Chonnam National University School of Medicine, Gwangju 61469, Republic of Korea; cecilyk@hanmail.net (Y.A.); myungho@chollian.net (M.H.J.)

**Keywords:** acute myocardial infarction, heart rate, cardiovascular outcomes

## Abstract

An elevated heart rate at admission or discharge is known to be associated with poor cardiovascular outcomes in patients with acute myocardial infarction (AMI). The association between post-discharge average office-visit heart rate and cardiovascular outcomes in patients with AMI has rarely been studied. We analyzed data for 7840 patients from the COREA-AMI registry who had their heart rates measured at least three times after hospital discharge. The office-visit heart rates were averaged and categorized into four groups by quartiles (<68, 68–74, 74–80, and >80 beats per minute). The primary end point was a composite of cardiovascular death, myocardial infarction, and ischemic stroke. During a median of 5.7 years of follow-up, major adverse cardiovascular events (MACE) affected 1357 (17.3%) patients. An average heart rate higher than 80 bpm was associated with an increased incidence of MACE compared to the reference average heart rate of 68–74 bpm. When dichotomized into <74 or ≥74 bpm, a lower average heart rate was not associated with MACE in patients with LV systolic dysfunction, in contrast to those without LV systolic dysfunction. An elevated average heart rate at office visits after AMI was associated with an increased risk of cardiovascular outcomes. Heart rate monitoring at office visits after discharge provides an important predictor related to cardiovascular events.

## 1. Introduction

Heart rate was determined to be a risk factor for mortality and morbidity in various cardiovascular diseases, including hypertension [1], heart failure [2], and coronary artery disease [1,3]. Heart rate is also a modifiable factor that can be controlled with medical treatments such as beta-blockers. During the early stage of acute myocardial infarction (AMI), an elevated heart rate at admission and discharge was associated with poor short- and long-term prognoses compared with a lower heart rate in patients treated with percutaneous coronary intervention (PCI) [4,5,6,7].

Guidelines recommend that beta-blocker treatment should be considered for patients with AMI, unless the patients are in Killip class III or higher, and have cardiogenic shock [8,9]. Beta-blockers reduce myocardial oxygen consumption by lowering heart rate, blood pressure, and myocardial contractility. However, many studies that investigated the long-term effect of beta-blockers in AMI and heart failure excluded patients with low heart rates (60–68 beats per min), with or without limitation of heart rate during dose escalation [10,11,12]. Moreover, there are reports that low doses of beta-blockers seem similarly effective as higher doses after AMI [13,14]. If achieving the target dose of beta-blockers is not critical, the question arises as to whether achieving a specific heart rate will determine the clinical outcomes. However, the threshold of heart rate at which risk increases in patients with AMI, and the relationship between increased heart rate and clinical events, are less well defined.

Thus, we sought to assess the association between heart rate during follow-up and cardiovascular outcomes in a real-world AMI registry to determine the optimal heart rate after acute myocardial infarction.

## 2. Materials and Methods

### 2.1. Patient Population and Data Collection

**Figure 1:**
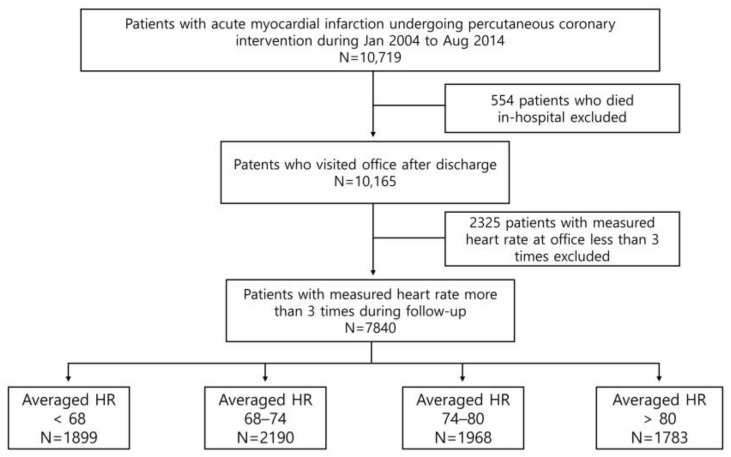
Study population. Flow chart outlining the selection of the study population.

Between January 2004 and April 2015, a total of 10,719 patients with AMI were registered in the Convergent Registry of Catholic and Chonnam University for Acute MI (COREA-AMI), which was designed to investigate real-world outcomes in all-comers with AMI. According to electrocardiography findings, the clinical diagnosis was divided into ST-segment elevation myocardial infarction (STEMI) and non-ST-segment elevation myocardial infarction (NSTEMI). The COREA-AMI I registry has been previously reported on, and has included patients with AMI who underwent PCI from January 2004 to December 2009. The COREA-AMI II registry added additional patients from January 2010 to August 2014. The current registry comprised updated new clinical and angiographic parameters and evaluated long-term clinical data for as long as possible until 2019. Patients were followed at 6 months after enrollment (PCI date), and every year thereafter. Their heart rates were collected from medical records of office visits, and were measured by automated blood pressure devices. The timing of the first office-visit heart rate measurement was 6 months after the index procedure (PCI), and every year thereafter. Figure 1 outlines the study flow and population. Of the 10,719 patients who presented with AMI, patients with heart rates that were measured less than 3 times (*n* = 2325), and those who died in the hospital (*n* = 554) were excluded. Thus, 7840 patients who had their heart rate measured at least 3 times after discharge were analyzed. Patients were categorized into four groups according to quartiles of average heart rate values.

### 2.2. PCI Procedure and Medical Treatment

Coronary angiography and primary percutaneous coronary intervention (PCI) were performed according to the current standard guidelines. Antiplatelet therapy and peri-procedural anticoagulation were administered according to standard regimens. All patients were prescribed aspirin (loading dose 200 mg) plus P2Y12 inhibitors, such as clopidogrel (loading dose 300 or 600 mg), ticagrelor (loading dose 180 mg) or prasugrel (loading dose 60 mg), before or during PCI. Predilation, direct stenting, postadditional balloon inflation, thrombus aspiration and administration of glycoprotein IIb/IIIa receptor blockers were performed at the discretion of individual interventional cardiologists. After the procedure, dual antiplatelet therapy was maintained for 1 year, and after that, the antiplatelet regimen was selected according to the balance of ischemic and bleeding risk. The necessity of beta-blocker treatment was decided by the physicians in each case. The dosage or the continuation of the use of beta-blocker treatment was left at the discretion of physicians. The post-intervention medications also included statins and renin-angiotensin-aldosterone blockers.

### 2.3. Study Endpoints and Definitions

The primary endpoint was a composite of cardiovascular death, myocardial infarction, and ischemic stroke. The secondary endpoints were individual components of the primary endpoint. All deaths were considered to be cardiovascular deaths unless a definite non-cardiac cause could be clearly identified. Myocardial infarction was defined as an elevated cardiac marker(s) with clinical evidence of ischemia, such as symptoms of myocardial ischemia, new ischemic electrocardiographic changes, and development of pathologic Q waves. Ischemic stroke was defined as the presence of a new focal neurologic deficit thought to be vascular in origin, with signs or symptoms lasting more than 24 h. For all composite outcomes, we analyzed the number of patients with at least one event from the composite outcomes. Patients with more than one contributing event were only counted once. The study protocol was approved by the ethics committee at each participating center, and was followed according to the principles of the Declaration of Helsinki. All patients provided written informed consent.

### 2.4. Statistical Analysis

Baseline characteristics are described as the mean ± standard deviation for continuous variables, and as the absolute number and percentage for categorical variables. Heart rates are expressed as a continuous variable, or averaged and categorized into four groups by quartiles (<68, 68–74, 74–80, and >80 beats per minute (bpm)). Differences in baseline characteristics between the different quartiles of average heart rate were evaluated using a one-way analysis of variance for continuous variables, and chi-square tests for categorical variables. Major adverse cardiovascular outcomes (MACE) were defined as cardiovascular death, myocardial infarction, and ischemic stroke. Multivariable analysis was performed to assess the prognostic value of an average heart rate and MACE after adjusting for sex, age, BMI, Killip class, hypertension, diabetes, previous MI, previous PCI, peripheral artery disease, end-stage renal disease, chronic lung disease, and left-ventricular systolic dysfunction (defined as a left-ventricular ejection fraction of less than 40%). Hazard ratios (HRs) were estimated with multivariable adjusted Cox proportional hazards models using the group with heart rates of 68–74 bpm as a reference. Survival curves were constructed with Kaplan–Meier estimates by groups for the primary composite end points and individual components of MACE. The groups were compared using the log-rank test for time-to-event data with respect to cardiovascular death, myocardial infarction, and ischemic stroke. Time-to-event was the duration between the index procedure (PCI) and the occurrence of the clinical events. The unadjusted and adjusted relationships between heart rate and primary outcomes were also assessed using a time-dependent Cox PH model with restricted cubic splines. We calculated hazard ratios for average heart rate (a heart rate of 74 bpm or greater) within each subgroup using Cox PH models. The cutoff of 74 bpm was selected as the median average heart rate in the study population.

All statistical tests were two-sided, and a *p* value of 0.05 was considered to be statistically significant. All statistical analyses were performed with R version 4.0.2 (R Foundation for Statistical Computing, Vienna, Austria).

## 3. Results

### 3.1. Baseline Characteristics

Among the 10,719 patients included in the COREA-AMI registry, data for 7840 patients with heart rate measured at least 3 times after hospital discharge were available. The baseline characteristics of the total patient population are shown in Table 1. The mean age at baseline was 61.9 ± 12.3 years, and most patients were men (5825 patients, 74.3%). The mean heart rates at admission and discharge were 78 ± 18.1 and 72 ± 8.6 bpm, respectively. The rate of beta-blocker prescription at discharge was 94.7%. Patients with beta-blockers at discharge had a lower average heart rate than those without β-blockers at discharge (73.7 ± 8.18 bpm vs. 75.6 ± 9.08 bpm; *p* < 0.001). The mean average heart rate at the office visit was 74 ± 8.42 bpm (interquartile range 68.09–79.29). When patients were categorized into four groups according to the quartiles of average heart rate, there was a significant trend toward a higher average heart rate with diabetes, chronic lung disease, high heart rate at admission and discharge, decreased LV systolic function, and low prescription rate of beta-blockers and renin-angiotensin-aldosterone blockers at discharge. There were no significant differences among the heart rate quartiles with regard to body mass index, hypertension, dyslipidemia, smoking status, end-stage renal disease, and peripheral artery disease. The prescription rate of beta-blockers decreased as the quartile of average heart rate increased (95.9% for a heart rate <68 bpm, 95.0% for a heart rate 68–74 bpm, 94.5% for a heart rate 74–80 bpm, and 93.2% for a heart rate > 80 bpm; *p* = 0.007).

### 3.2. Average Heart Rate and Cardiovascular Outcome

During a median follow-up period of 5.7 years (IQR 3.8–7.6), the primary outcome, which is a composite of cardiovascular death, myocardial infarction, and ischemic stroke, occurred in 1357 patients (17.3%). For the secondary outcomes, cardiovascular death, myocardial infarction, and ischemic stroke occurred in 781 (10.0%), 474 (6.0%), and 272 (3.5%) patients, respectively (Table 2).

**Figure 2:**
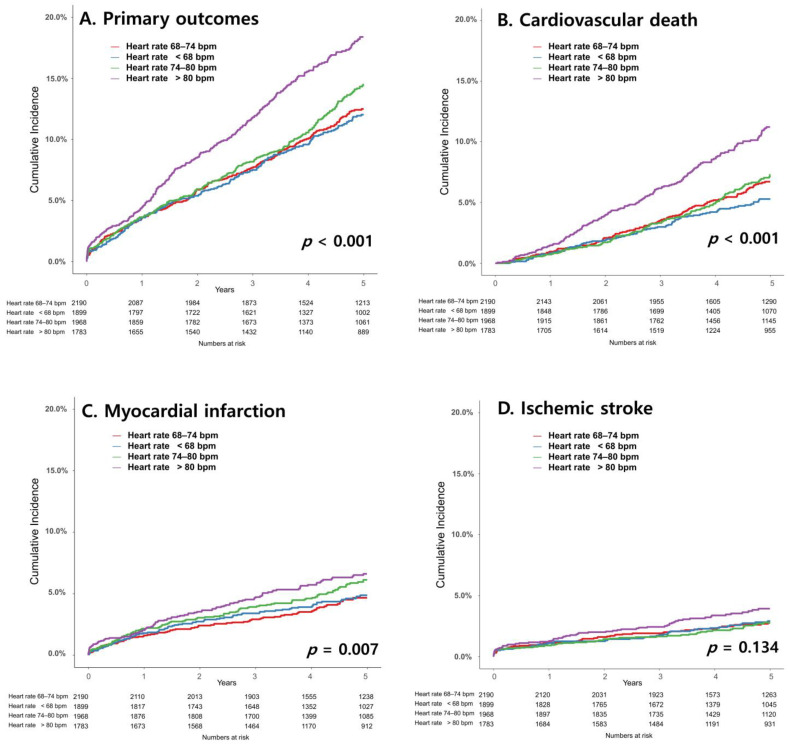
Kaplan–Meier curves for major adverse cardiovascular events (**A**), cardiovascular death (**B**), myocardial infarction (**C**), and ischemic stroke (**D**) according to quartiles of average office-visit heart rate.

Kaplan–Meier curves for average heart rate divided by quartiles and primary outcomes during the follow-up period are shown in Figure 2. Patients in the highest quartile, who had an average heart rate higher than 80 bpm, showed significantly higher event rates for cardiovascular death (14.6% vs. 9.0%) and myocardial infarction (7.5% vs. 5.2%) than patients in the reference quartile, who had an average heart rate of 68–74 bpm. The occurrence of ischemic stroke was not significantly different among the quartiles.

**Figure 3:**
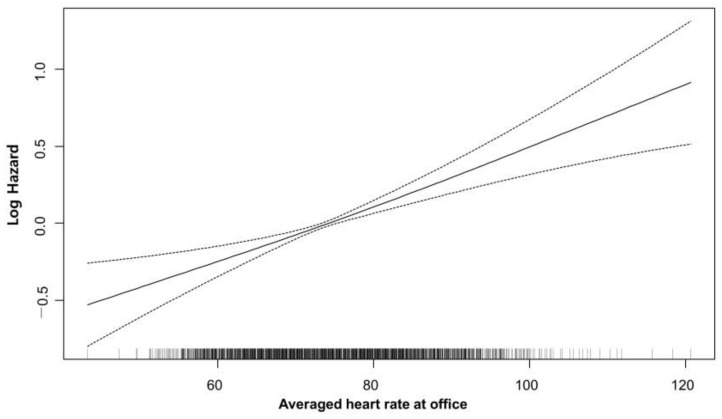
Restricted cubic spline curve of the primary outcome versus average office-visit heart rate.

Average heart rate was an independent predictor of major adverse cardiovascular events (Crude HR 1.025, 95% CI 1.02–1.03, *p* < 0.001). Discharge heart rate was not associated with the risk of MACE in univariate analysis (Crude HR 1.001, 95% CI 0.999–1.012, *p* = 0.082). Compared with the reference group (heart rate 68–74 bpm), the adjusted hazard ratio for the primary outcomes was 0.91 (95% CI 0.77–1.07, *p* = 0.246) for the heart rate < 68 bpm group, 1.12 (95% CI 0.96–1.3, *p* = 0.135) for the heart rate of 74–80 bpm group, and 1.39 (95% CI 1.2–1.61, *p* < 0.0001) for the heart rate higher than 80 bpm group. (Table 3) Patients with an average heart rate >80 bpm had a significantly higher risk for major adverse cardiovascular events (adjusted HRs 1.39, 95% CI 1.2–1.61, *p* < 0.001). In addition, age, BMI, Killip class, hypertension, diabetes, and LV systolic dysfunction were all independently associated with the risk of MACE in patients with AMI who were treated with PCI. Even after applying a restricted cubic spline Cox regression analysis, average heart rate showed a positive linear relationship with the risk of primary outcomes (Figure 3).

### 3.3. Subgroup Analysis

**Figure 4:**
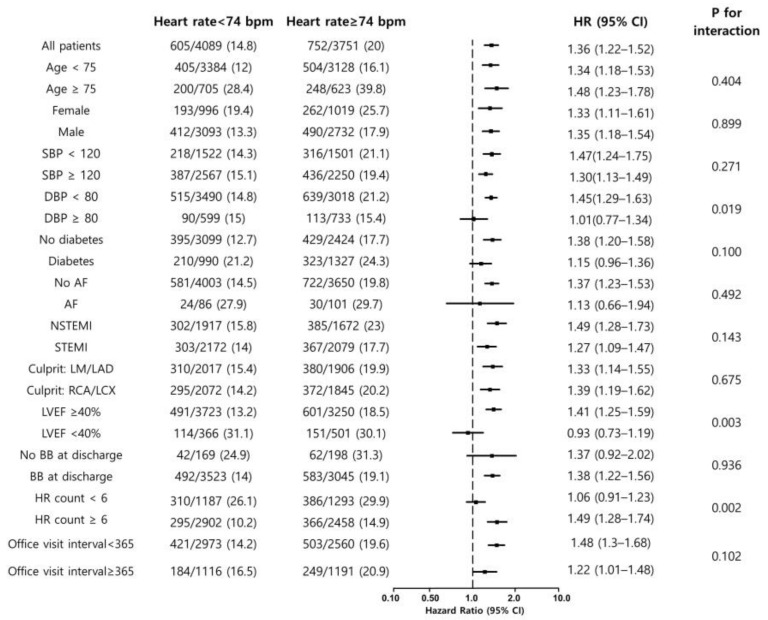
Subgroup analysis.

The average heart rate for the office visits showed significant interactions with LV systolic dysfunction and the number of heart rate measurements (Figure 4). Lower average heart rate was not associated with the incidence of primary outcomes in patients with LV systolic dysfunction in contrast to those without LV systolic dysfunction. Patients with LV systolic dysfunction were older and had more hypertension, diabetes, prior myocardial infarction, prior PCI, low renal function and more cardiogenic shock (Supplementary Table S1). In patients with severe LV systolic dysfunction, the average heart rate was higher (73.8 ± 8.3 vs. 76.3 ± 8.7 bpm, *p* < 0.001), and the prescription rate of β-blockers was lower. In addition, a higher average heart rate was associated with MACE in patients with heart rate measurements recorded on more than six occasions. The survival period was longer in the group of patients with heart rate measurements recorded on more than six occasions (1748.5 ± 1074.1 vs. 2283.9 ± 870.0 days, *p* < 0.001).

## 4. Discussion

The major findings of this study regarding average heart rate and clinical outcomes of acute myocardial infarctions undergoing PCI were as follows: (1) A higher average office-visit heart rate was associated with an increased risk of cardiovascular events in the post-MI period. (2) Cardiovascular mortality and myocardial infarction increased in patients with a higher average heart rate. (3) Average office-visit heart rate had a linear relationship with cardiovascular events. The lower the average heart rate was, the better the clinical outcomes experienced during the post-MI period.

In patients with AMI, there were results showing a link to prognosis depending on the heart rate at admission and discharge. Perne et al. suggested that a heart rate >90 bpm at admission reduced survival at 3 months in patients presenting with myocardial infarction in a large German registry [15]. Antoni et al. showed that a heart rate ≥ 70 bpm had a twofold increased risk of cardiovascular mortality in ST-segment elevation AMI [6]. Seronde et al. and Alapti et al. also documented consistent results that indicated a higher discharge heart rate was associated with poor cardiovascular mortality [5,16]. Because heart rate can change as cardiac function recovers after stabilization, heart rate after discharge could be the actual treatment target to be adjusted in long-term care. However, fewer investigators have examined the association of office-visit heart rate, a potentially modifiable therapeutic target, with post-AMI outcomes. To our knowledge, the present study is the first and largest study to evaluate the association of average heart rate at office visits with outcomes after AMI. Our study demonstrated that the average heart rate at office visits is a useful predictor of long-term cardiovascular outcomes in the stable post-AMI period. Contrary to the previous study, there was no association between discharge heart rate and long-term prognosis. This study demonstrates the importance of keeping tight control of post-discharge office-visit heart rate in patients with AMI.

The oldest biomarker in cardiovascular medicine is resting heart rate. Heart rate is a major determinant of myocardial oxygen demand, and it affects coronary blood flow through diastolic filling time. The prognostic value of heart rate in patients with various heart diseases, including myocardial infarction, hypertension, and heart failure, is well established [17,18,19]. The association between elevated heart rate on admission and CV mortality during AMI has been recognized and incorporated into different risk prediction models, such as the GRACE [20] and the TIMI risk score [4]. In an earlier study analyzing the COREA-AMI registry, Choo et al. reported that beta-blocker treatment was associated with reduced long-term mortality in patients with acute MI [21]. Consequently, drugs that control heart rate, i.e., β-blockers, have long been mandatory for AMI patients because elevated heart rate has poor clinical outcomes in post-MI treatment [8,9,22]. Although guidelines recommended the use of beta-blockers in patients with myocardial infarction based on previous trials [10,11,12], there is still debate among clinicians regarding the optimal dosing strategy for β-blockers in this patient population. Specifically, there is uncertainty regarding whether a target dose or a target heart rate is more important for achieving optimal outcomes. The target dose strategy aims to achieve a certain dose of β-blocker in patients with AMI, typically based on the dose used in clinical trials. This approach is based on the premise that higher doses of β-blockers are associated with better outcomes. Several large randomized controlled trials have demonstrated that high doses of β-blockers are associated with a reduction in mortality and reinfarction in patients with AMI [10,23]. However, achieving the target dose of β-blockers can be challenging in clinical practice, as some patients may not tolerate high doses due to side effects, such as bradycardia, hypotension, and fatigue. Additionally, elderly patients and those with comorbidities may be more susceptible to adverse effects. Thus, European guidelines limit the heart rate to 55–60 bpm at rest to avoid the side effects of β-blockers [9]. A study-level meta-regression analysis showed a significant association between a reduction in heart rate and decreased cardiac mortality [24]. Our study demonstrated that the achieved heart rate at office visits correlated positively and linearly with the risk of cardiovascular event rates. Additionally, the average heart rate during follow-up was correlated with the prescription rate of β-blockers in our study. This finding is consistent with a recent study showing that the associations between heart rate at the first outpatient visit and MACE were approximately linear [25]. There are reports demonstrating no significant additional benefit of high-dose β-blockers compared with low-dose β-blockers [14,26]. Therefore, when we prescribe β-blockers for patients with myocardial infarction, the titrate of β-blockers would be based on the achieved heart rate rather than the target dose of β-blockers.

In heart failure, elevated heart rate is considered a physiological and beneficial compensatory response up to a certain point, whereas there might be a direct causative effect on outcomes in coronary disease. Heart rate (measured at rest and during sinus rhythm) has prognostic importance in patients with left-ventricular systolic dysfunction, and changes in heart rate are predictive of outcomes [27]. In contrast, a reduction in resting heart rate in patients with stable coronary artery disease and left-ventricular systolic dysfunction was associated with a significant reduction in cardiovascular outcomes in those with heart rates above 70 bpm [28]. Meanwhile, in our subgroup analysis, a lower average heart rate was not associated with clinical outcomes in patients with AMI and left-ventricular systolic dysfunction. This may be due to the low number of patients, the insufficient number of heart-rate measurements at the office due to high mortality and morbidity, or the failure to maintain a heart rate low enough to improve the clinical outcome in the low heart-rate group.

Our study had several limitations. First, the possibility of unobserved confounders and selection bias should be considered because our study was retrospective, observational, and nonrandomized. Second, we did not analyze the type, dose, and achievement rate of the recommended dose of β-blockers. The results may be impacted by these factors. However, in the OBTAIN and KAMIR studies, there was no correlation between the dose of β-blockers and the survival benefit [13,14]. Given these results, it can be assumed that the office-visit heart rate was a more important factor affecting clinical outcomes. Third, the COREA-AMI registry was a very long-term study. Over the 10-year period, there were many changes in the treatment of post-AMI patients, and the addition of techniques such as stents and devices. These changes may have influenced the results.

## 5. Conclusions

These large, real-world data show that elevated average office-visit heart rate is associated with poor clinical outcomes in patients with acute myocardial infarction. Heart rate monitoring at post-discharge office visits provides an important predictor related to cardiovascular events. A higher office heart rate was a risk factor associated with cardiovascular death and recurrent myocardial infarction. Therefore, the thorough tracking of heart-rate changes in outpatients can aid the treatment of patients in the post-MI period.

## Figures and Tables

**Table 1:** Baseline characteristics.

	Total (*n* = 7840)	<68 bpm (*n* = 1899)	68–74 bpm (*n* = 2190)	74–80 bpm (*n* = 1968)	>80 bpm (*n* = 1783)	*p* Value
Demographic						
Age, y	61.9 ± 12	63 ± 12	62 ± 12	61 ± 13	62 ± 12	<0.001
Male sex, *n* (%)	5825 (74.3%)	1463 (77.0%)	1630 (74.4%)	1445 (73.4%)	1287 (72.2%)	0.006
Initial BMI, kg/m^2^	24.33 ± 3.19	24.26 ± 3.04	24.44 ± 3.17	24.38 ± 3.14	24.21 ± 3.42	0.097
Hypertension, *n* (%)	3947 (50.3%)	969 (51.0%)	1109 (50.6%)	972 (49.4%)	897 (50.3%)	0.766
Diabetes mellitus, *n* (%)	2317 (29.6%)	397 (20.9%)	593 (27.1%)	604 (30.7%)	723 (40.5%)	<0.001
Hypercholesterolemia, *n* (%)	1343 (17.1%)	341 (18.0%)	388 (17.7%)	338 (17.2%)	276 (15.5%)	0.182
Currently smoke, *n* (%)	3319 (42.3%)	783 (41.2%)	890 (40.6%)	854 (43.4%)	792 (44.4%)	0.055
Family history of CAD, *n* (%)	240 (3.1%)	55 (2.9%)	70 (3.2%)	64 (3.3%)	51 (2.9%)	0.851
Prior MI	291 (3.7%)	2 (2.8%)	84 (3.8%)	78 (4.0%)	76 (4.3%)	0.091
Prior CABG	36 (0.5%)	8 (0.4%)	10 (0.5%)	8 (0.4%)	10 (0.6%)	0.900
Prior PCI	512 (6.5%)	113 (6.0%)	142 (6.5%)	129 (6.6%)	128 (7.2%)	0.516
Prior stroke, *n* (%) (or CVA)	496 (6.3%)	113 (6.0%)	135 (6.2%)	120 (6.1%)	128 (7.2%)	0.406
Atrial arrhythmia, *n* (%)	187 (2.4%)	40 (2.1%)	46 (2.1%)	46 (2.3%)	55 (3.1%)	0.16
Peripheral arterial disease, *n* (%)	38 (0.5%)	11 (0.6%)	9 (0.4%)	8 (0.4%)	10 (0.6%)	0.786
Chronic lung disease, *n* (%) (or COPD)	154 (2.0%)	28 (1.5%)	34 (1.6%)	31 (1.6%)	61 (3.4%)	<0.001
Chronic renal failure, *n* (%)	118 (1.5%)	25 (1.3%)	30 (1.4%)	27 (1.4%)	36 (2.0%)	0.247
Cancer, *n* (%)	254 (3.2%)	56 (2.9%)	60 (2.7%)	68 (3.4%)	69 (3.8%)	0.185
Clinical						
Initial ECG diagnosis as STEMI, *n* (%)	4258 (54.3%)	988 (52.0%)	1189 (54.3%)	1102 (56.0%)	979 (54.9%)	0.076
Initial systolic BP, mmHg	129.2 ± 26.4	129.9 ± 26.3	129.6 ± 25.8	129.2 ± 27.0	127.9 ± 26.6	0.123
Initial diastolic BP, mmHg	78.9 ± 16.3	78.9 ± 15.9	78.9 ± 16.0	79.0 ± 16.8	78.8 ± 16.8	0.966
Heart rate at admission, b.p.m	78.9 ± 16.3	73.1 ± 16.7	76.7 ± 17.0	79.6 ± 18.2	83.3 ± 19.2	<0.001
Heart rate at discharge, b.p.m	72 ± 8.6	67 ± 7.3	70.9 ± 7.2	73.2 ± 7.5	77.2 ± 9.3	<0.001
Killip class, *n* (%)						0.001
I	5720 (79.7%)	1452 (82.0%)	1628 (80.7%)	1403 (78.3%)	1237 (77.2%)	
II	573 (8.0%)	133 (7.5%)	166 (8.2%)	145 (8.1%)	129 (8.1%)	
III	365 (5.1%)	68 (3.8%)	87 (4.3%)	99 (5.5%)	111 (6.9%)	
IV	522 (7.3%)	117 (6.6%)	136 (6.7%)	144 (8.0%)	125 (7.8%)	
eGFR, mL/min per 1.73 m^2^	79.1 ± 24.0	79.6 ± 23.1	80.1 ± 23.3	79.8 ± 23.9	76.7 ± 25.8	<0.001
CK-MB, peak	121.53 ± 225	117 ± 181	114 ± 165	128 ± 321	128 ± 204	0.098
LV systolic function, EF (%)	53.8 ± 10.8	55.6 ± 9.9	54.5 ± 10.9	53.2 ± 10.7	51.7 ± 11.1	<0.001
Cardiogenic shock	232 (3.0%)	52 (2.7%)	69 (3.2%)	61 (3.1%)	50 (2.8%)	0.830
Culprit vessel						
Left main	205 (2.6%)	44 (2.3%)	56 (2.6%)	45 (2.3%)	60 (3.4%)	
LAD	3718 (47.4%)	888 (46.8%)	1029 (47.0%)	940 (47.8%)	861 (48.3%)	
LCX	1324 (16.9%)	328 (17.3%)	356 (16.3%)	342 (17.4%)	298 (16.7%)	
RCA	2578 (32.9%)	634 (33.4%)	745 (34.0%)	639 (32.5%)	560 (31.4%)	
Total stent number	1.56 ± 0.8	1.5 ± 0.9	1.6 ± 0.9	1.5 ± 0.9	1.6 ± 1.0	0.003
Mean stent diameter	3.17 ± 0.41	3.2 ± 0.4	3.2 ± 0.4	3.2 ± 0.4	3.2 ± 0.4	0.032
Total stent length	34.0 ± 20.7	32.4 ± 19.5	34.1 ± 20.7	34.0 ± 20.5	35.7 ± 22.3	<0.001
Discharge medications						
Aspirin	7737 (98.7%)	1881 (99.1%)	2162 (98.7%)	1940 (98.6%)	1754 (98.4%)	0.320
P2Y12 inhibitor	7015 (89.4%)	1896 (99.8%)	2185 (99.7%)	1964 (99.7%)	1771 (99.3%)	0.271
Beta-blocker	6568 (94.7%)	1639 (95.9%)	1884 (95.0%)	1644 (94.5%)	1401 (93.2%)	0.007
RAA blocker	6168 (78.6%)	1547 (81.5%)	1766 (80.6%)	1560 (79.3%	1295 (72.6%)	<0.001
Statin	7411 (94.5%)	1762 (97.6%)	2015 (97.0%)	1811 (97.1%)	1599 (96.1%)	0.070
Follow-up						
Follow-up mean systolic BP, mm Hg	123.0 ± 10.7	123.7 ± 10.8	123.2 ± 10.5	123.2 ± 10.6	122.1 ± 11.1	<0.001
Follow-up mean diastolic BP, mm Hg	73.2 ± 7.1	72.1 ± 7.1	73.2 ± 6.9	74.0 ± 7.1	73.7 ± 7.2	<0.001
Averaged heart rate at office, b.p.m	74.03 ± 8.42	63.8 ± 3.4	71.0 ± 1.7	76.7 ± 1.7	85.7 ± 5.2	<0.001
Heart rate measurement count	7.1± 3.0	7.2 ± 3.1	7.4 ± 3.1	7.2 ± 3.0	6.7 ± 3.1	<0.001
Heart rate measurement interval, days	349.5 ± 268.9	350.6 ± 267.8	320.7 ± 231.5	352.7 ± 270.6	373.2 ± 306.0	<0.001

BMI, body mass index; CAD, coronary artery disease; MI, myocardial infarction; CABG, coronary artery bypass surgery; PCI, percutaneous coronary intervention; ECG, electrocardiogram; BP, blood pressure; bpm, beats per minute; RAA, renin-angiotensin-aldosterone.

**Table 2:** Major adverse cardiovascular events.

	Total (*n* = 7840)	<68 bpm (*n* = 1899)	68–74 bpm (*n* = 2190)	74–80 bpm (*n* = 1968)	>80 bpm (*n* = 1783)	*p* Value
MACE	1357 (17.3%)	261 (13.7%)	344 (15.7%)	345 (17.5%)	407 (22.8%)	<0.0001
Cardiovascular death	781 (10.0%)	127 (6.7%)	197 (9.0%)	196 (10.0%)	261 (14.6%)	<0.0001
Myocardial infarction	474 (6.0%)	102 (5.4%)	113 (5.2%)	126 (6.4%)	133 (7.5%)	0.010
Ischemic stroke	272 (3.5%)	59 (3.1%)	72 (3.3%)	64 (3.3%)	77 (4.3%)	0.166

MACE, major adverse cardiovascular events.

**Table 3:** Multivariable analysis of major adverse cardiovascular events over 5 years in patients with acute myocardial infarction.

	Crude HR (95%CI)	Crude *p* Value	Adj. HR (95%CI)	Adj. *p* Value
Averaged heart rate at office (bpm)				
68–74	Reference			
<68	0.91 (0.77–1.07)	0.242	0.91 (0.77–1.07)	0.246
74–80	1.13 (0.97–1.31)	0.116	1.12 (0.96–1.3)	0.135
>80	1.51 (1.31–1.74)	<0.001	1.39 (1.2–1.61)	<0.001
Age	1.05 (1.05–1.06)	<0.001	1.04 (1.04–1.05)	<0.001
Body mass index	0.93 (0.91–0.95)	<0.001	0.98 (0.96–0.99)	0.009
Killip class				
1	Reference			
2	1.64 (1.36–1.97)	<0.001	1.27 (1.05–1.52)	0.013
≥3	1.81 (1.56–2.09)	<0.001	1.28 (1.1–1.49)	0.001
Hypertension	1.67 (1.5–1.87)	<0.001	1.2 (1.07–1.35)	0.002
Diabetes	1.69 (1.51–1.88)	<0.001	1.31 (1.17–1.46)	<0.001
Previous MI	2.13 (1.72–2.62)	<0.001	1.33 (1.04–1.71)	0.024
Previous PCI	2.03 (1.7–2.41)	<0.001	1.39 (1.13–1.71)	0.002
Peripheral artery disease	2.53 (1.5–4.29)	<0.001	1.97 (1.16–3.35)	0.012
End stage renal disease	5.26 (4.08–6.79)	<0.001	3.74 (2.87–4.86)	<0.001
Chronic lung disease	2.05 (1.53–2.75)	<0.001	1.34 (0.99–1.8)	0.054
LV dysfunction	2.21 (1.93–2.52)	<0.001	1.57 (1.36–1.8)	<0.001

MI, myocardial infarction; PCI, percutaneous coronary intervention; LV, left ventricle.

## Data Availability

The data presented in the current study are available on reasonable request from the corresponding author.

## Supplementary Materials

The following supporting information can be downloaded at: https://www.mdpi.com/article/10.3390/jcm12113734/s1, Table S1: Baseline characteristics according to left ventricular systolic function.
