# Supplementary material for: Office-Visit Heart Rate and Long-Term Cardiovascular Events in Patients with Acute Myocardial Infarction
Source: J Clin Med. 2023 May 29;12(11):3734. doi: 10.3390/jcm12113734 (PMC10253943; doi:10.3390/jcm12113734)
Supplement: Supplementary file 1 [file jcm-12-03734-s001.zip › jcm-2379919-supplementary.pdf]

Office-Visit Heart Rate and Long-term Cardiovascular Events in Patients with Acute Myocardial Infarction

Supplementary Table S1. Baseline characteristics according to left ventricular systolic function

	Averaged heart rate at office (Total=7,840)		
**Covariates**	**LVEF ≥ 40 n=6973**	**LVEF < 40 n=867**	**P value**
**Demographic**			
Age, y	61.5±12.2	65.5±12.7	<0.001
Male sex, n (%)	5213 (74.8)	612 (70.6)	0.009
Initial BMI, kg/m2	24.4±3.2	23.7±3.2	<0.001
Hypertension, n (%)	3479 (49.9)	468 (54.0)	0.025
Diabetes mellitus, n (%)	1972 (28.3)	345 (39.8)	<0.001
Hypercholesterolemia, n (%)	1212 (17.4)	131 (15.1)	0.104
Current smoke, n (%)	2991 (42.9)	328 (37.8)	0.005
Family history of CAD, n(%)	220 (3.2)	20 (2.3)	0.207
Prior MI	230 (3.3)	61 (7.0)	<0.001
Prior CABG	31 (0.4)	5 (0.6)	0.59
Prior PCI	422 (6.1)	90 (10.4)	<0.001
Prior stroke, n (%) (or CVA)	431 (6.2)	65 (7.5)	0.153
Peripheral arterial disease, n (%)	35 (0.5)	3 (0.3)	0.794
Chronic lung disease, n (%) (or COPD)	120 (1.7)	34 (3.9)	<0.001
Chronic renal failure, n (%)	93 (1.3)	25 (2.9)	0.001
Cancer, n (%)	219 (3.1)	34 (3.9)	0.261
**Clinical**			
Initial ECG diagnosis as STEMI, n (%)	3750 (54.0)	508 (59.1)	0.005
Initial systolic BP, mm Hg	129.4 ± 26.4	128.0 ± 27.1	0.178
Initial diastolic BP, mm Hg	79.0 ± 16.3	78.9 ± 17.2	0.846
Heart rate at admission, bpm	76.8 ± 17.2	88.7 ± 21.6	<0.001
Killip class, n (%)			<0.001
I	5829 (83.6)	551 (63.6)	
II	480 (6.9)	93 (10.7)	
III	217 (3.1)	148 (17.1)	
IV	447 (6.4)	75 (8.7)	
eGFR, mL/min per 1.73 m2	80.6 ± 23.5	67.9 ± 25.3	<0.001
LV systolic function, EF(%)	56.4 ± 8.1	33.7 ± 5.6	<0.001

Cardiogenic shock	184 (2.6)	48 (5.5)	<0.001
**Discharge medications and follow up**			
Aspirin	6886 (98.8)	851 (98.2)	0.194
Beta-blocker	5869 (95.0)	699 (92.5)	0.005
Statin	6410 (96.9)	777 (97.2)	0.718
Follow up mean systolic BP, mm Hg	123.4±10.5	120.4±12.4	<0.001
Follow up mean diastolic BP, mm Hg	73.5±7.1	71.3±7.5	<0.001
Averaged heart rate at office, bpm	73.8±8.3	76.3±8.7	<0.001
Heart rate measurement count	7.2±3.1	6.7±3.2	<0.001

BMI, body mass index; CAD, coronary artery disease; MI, myocardial infarction; CABG, coronary artery bypass surgery; PCI, percutaneous coronary intervention; ECG, electrocardiogram; BP, blood pressure; bpm, beats per minute; RAA, renin-angiotensin-aldosterone;
